# A Systematic Review of Mortality Rates Among Adult Acute Respiratory Distress Syndrome Patients Undergoing Extracorporeal Membrane Oxygenation Therapy

**DOI:** 10.7759/cureus.43590

**Published:** 2023-08-16

**Authors:** Raghavendra R Sanivarapu, Usama Osman, Abishek Latha Kumar

**Affiliations:** 1 Pulmonary and Critical Care Medicine, Texas Tech University Health Sciences Center, Lubbock, USA; 2 Internal Medicine, California Institute of Behavioral Neurosciences and Psychology, Fairfield, USA; 3 Research, California Institute of Behavioral Neurosciences and Psychology, Fairfield, USA; 4 Geriatrics, Michigan State University College of Human Medicine, East Lansing, USA

**Keywords:** extracorporeal membrane oxygenation support, mortality rate in icu, va-ecmo, vv-ecmo, ards (acute respiratory distress syndrome)

## Abstract

Acute respiratory distress syndrome (ARDS) is a severe lung disease associated with a high mortality rate. Extracorporeal membrane oxygenation (ECMO) is a life-saving therapy for severe ARDS patients who do not respond to conventional treatments. Nevertheless, the optimal management of ARDS patients undergoing ECMO and their mortality rates remain subjects of controversy. Thus, this systematic review aims to assess mortality rates in ARDS patients on ECMO and identify associated factors. The review adhered to the Preferred Reporting Items for Systemic Review and Meta-Analysis (PRISMA) 2020 guidelines. A comprehensive literature search was conducted on PubMed, PubMed Central (PMC), Medline, and Embase. In accordance with our inclusion and exclusion criteria, filters, and key terms, we proceeded to screen the articles. After assessing the relevance of each article to our topic, further screening was carried out. Quality assessment of the articles was conducted, resulting in the inclusion of a total of 12 articles for the review. The primary outcome focused on mortality rates among ARDS patients undergoing ECMO. Secondary outcomes explored potential contributors to mortality, including patient age, underlying cause of ARDS, and Sequential Organ Failure Assessment (SOFA) scores at the initiation of ECMO. Mortality rates exhibited significant variation, ranging from 22% to 62.6%. Several factors emerged as potential predictors of mortality, encompassing patient age, comorbidities, complications during ECMO therapy, and treatment-related variables. This systematic review offers valuable insights into the intricate factors influencing mortality rates among ARDS patients on ECMO. A comprehension of these factors is essential to steer clinical practice and enhance patient outcomes. While ECMO serves as a restorative avenue for ARDS patients, future research is warranted to further elucidate these complex interactions and refine ECMO therapy protocols.

## Introduction and background

Acute respiratory distress syndrome (ARDS) is a severe lung disease characterized by inflammation, pulmonary edema, and respiratory failure, which can lead to hypoxemia and multi-organ failure [1]. The incidence of ARDS is estimated to be 10-86 cases per 100,000 people per year, with a mortality rate ranging from 30% to 60% [2,3]. Despite advances in critical care management, ARDS remains a significant health problem worldwide, and mortality rates in severe cases remain high [4].

Extracorporeal membrane oxygenation (ECMO) is a life-saving therapy for patients with severe ARDS who do not respond to conventional treatment [5]. ECMO provides respiratory and circulatory support by temporarily replacing the function of the heart and lungs. The therapy involves using a mechanical pump to circulate blood through an artificial lung (oxygenator), which removes carbon dioxide and adds oxygen before returning the blood to the patient's body [6]. ECMO can provide a bridge to recovery for ARDS patients, allowing the lungs time to heal while supporting oxygenation and ventilation [7].

The use of ECMO in ARDS patients has increased in recent years due to technological advancements and improvements in patient selection and management. However, the optimal management of ARDS patients on ECMO remains controversial, and mortality rates in these patients vary widely across studies [8]. In addition, several factors can affect mortality rates in ARDS patients on ECMO, including patient selection, the timing of ECMO initiation, duration of ECMO support, and complications associated with ECMO use [9].

A systematic review of the literature can provide insight into the mortality rates of ARDS patients on ECMO and identify factors associated with mortality. Several previous systematic reviews have evaluated mortality rates in ARDS patients on ECMO. For example, Zampieri et al. in 2013 published a systematic review that reported a pooled mortality rate of 43% in ARDS patients on ECMO [10]. Another systematic review reported a mortality rate of 38% in ARDS patients on ECMO [11]. However, these reviews included studies with varying patient populations and ECMO management protocols, which may have affected the results.

Therefore, there is a need for an updated systematic review that includes only studies with a uniform patient population and standardized ECMO management protocols. This review aims to evaluate the mortality rates of ARDS patients on ECMO in recent studies and identify factors associated with mortality. The review will provide valuable information for clinicians caring for ARDS patients on ECMO and guide future research.

## Review

Methodology

This systemic review followed the Preferred Reporting Items for Systemic Review and Meta-Analysis (PRISMA) 2020 guidelines [12].

Search Sources and Strategy

We searched PubMed, PubMed Central (PMC), Medline, and Embase for the relevant literature. We used various combinations of ARDS, ECMO, and mortality to search all databases. In PubMed, however, along with these keywords, the following strategy was developed and used to search relevant literature in PubMed’s MeSH database: ((“Extracorporeal Membrane Oxygenation”[Mesh] OR “Oxygenators, Membrane”[Mesh]) AND (“Respiratory Distress Syndrome, Adult”[Mesh] OR “Respiratory Insufficiency”[Mesh])) OR ((“Extracorporeal Membrane Oxygenation”[Mesh] OR “Oxygenators, Membrane”[Mesh]) AND “Respiratory Insufficiency”[Mesh] AND (“Carbon Dioxide”[Mesh] OR “Hypercapnia”[Mesh])) AND “humans”[MeSH Terms] AND (“adolescent”[MeSH Terms] OR “adult”[MeSH Terms]). 

Inclusion and Exclusion Criteria

We selected articles from the latest literature published in the past ten years, including papers written in the English language, or if the full-text English-language translation is available. We only included research papers involving human participants and studies done only in adults aged more than 19 years. We screened articles that are randomized controlled studies and observational studies alone, and case series and case reports were excluded. We included patients both on veno-venous (VV) ECMO and veno-arterial (VA) ECMO. Articles were excluded if the full text of the papers could not be retrieved. Articles not involving mortality data were excluded, and articles with an n value of less than 50 patient data were excluded as well. Gray literature and proposal papers were also not included.

Selection Process

We used the Endnote application in screening the articles. Duplicate articles were removed. Each article was thoroughly screened by two authors for eligibility. The concerns were discussed with all co-authors and finalized. The final screened articles were further evaluated by adding inclusion and exclusion criteria and those that satisfied the criteria were finalized.

Quality Assessment of the Studies

The finalized articles were thoroughly checked for quality utilizing relevant quality appraisal tools. All co-authors were involved in quality checks. Observational studies were assessed for quality using the Newcastle-Ottawa tool. Articles with high quality were included in the systematic review. The articles were assessed for eligibility using the relevant quality appraisal tools. Only one study was a randomized control trial, which was included and the Cochrane bias assessment tool was utilized in its quality assessment. Table 1 shows the results of the quality appraisal for the observational studies included in the review.

**Table 1:** Quality appraisal of articles using the Newcastle-Ottawa tool.

Author	Year	Selection	Comparability	Outcome
Roch et al. [13]	2014	***	*	**
Haneya et al. [14]	2015	***	*	**
Schmidt et al. [15]	2015	****	*	**
Burrell et al. [16]	2017	****	**	**
Na et al. [17]	2019	***	*	**
Lim et al. [18]	2019	***	*	**
Galvagno et al. [19]	2020	****	*	**
Warren et al. [20]	2020	****	**	**
Seeliger et al. [21]	2021	***	*	**
Urner et al. [22]	2022	****	**	**
Warren et al. [23]	2022	****	**	**

Data Collection

The final articles were thoroughly reviewed, and the primary outcome of mortality was extracted. Also, other relevant information was gathered. All authors were involved in the extraction of data and finalizing the results.

Results

Study Identification and Selection

**Figure 1:**
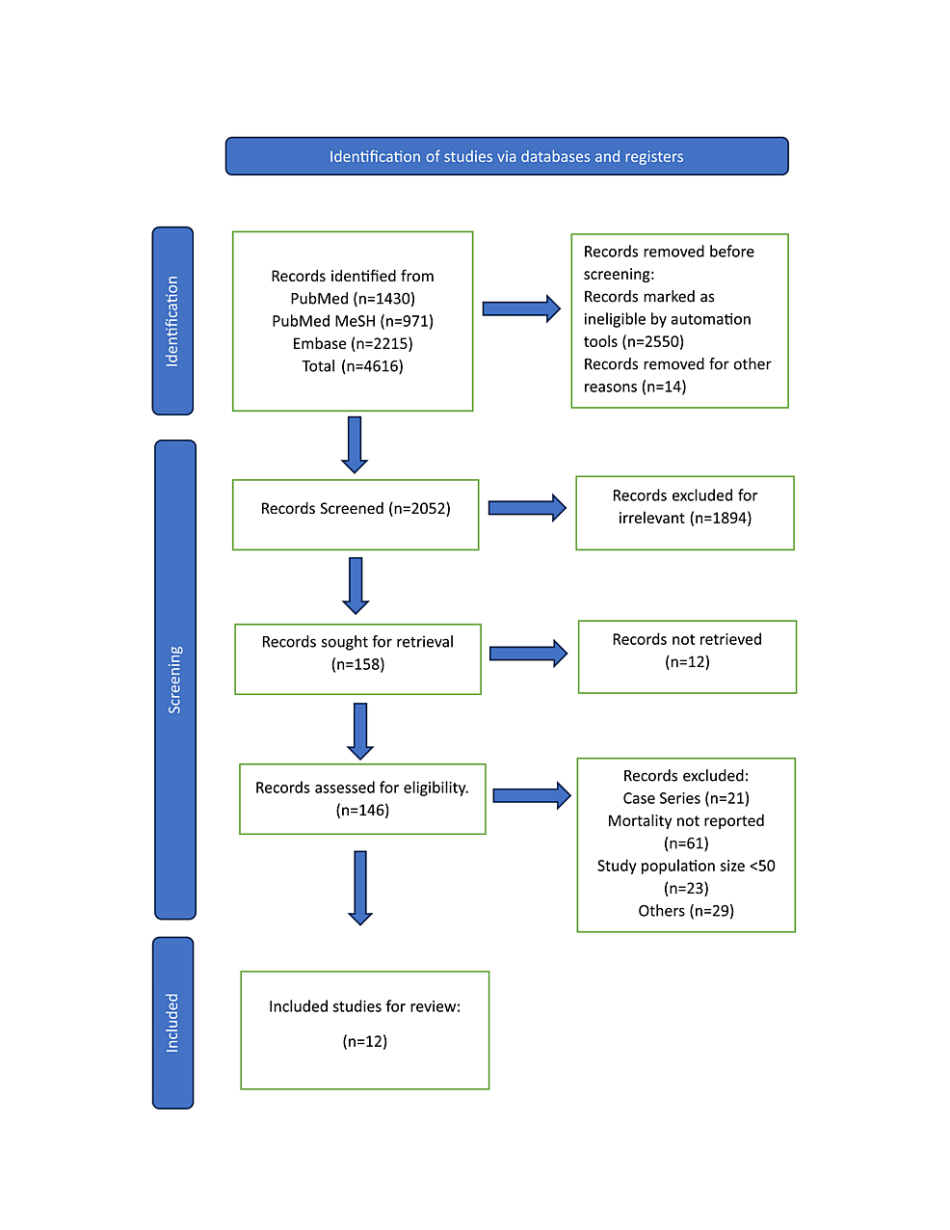
PRISMA 2020 flow diagram for systematic review

A literature search in different databases yielded a total of 4616 relevant articles. In total, 2550 articles were removed by automation tool applying, the published articles in the past 10 years, English language, and studies that are relevant only for adults aged 19 years and above. A total of 2052 articles were screened and assessed for eligibility by going through the titles and abstracts. We included only observational studies and randomized control trials (RCT) and a total of 146 articles were shortlisted. We removed case series and studies with n value less than 50 to avoid confounding factors and 12 articles were finalized for review. The selection process of the studies is shown in Figure 1 in the PRISMA flowchart.

Outcomes Measured

The primary outcome focused in this systematic review is mortality rates in patients with ARDS on ECMO. The secondary outcomes that were looked at are factors that could have potentially contributed to mortality. The rate of renal replacement therapy (RRT) while receiving ECMO was looked at as well. The other factors included were age, cause of ARDS, and Sequential Organ Failure Assessment (SOFA) scores at ECMO initiation. The outcomes from each study are tabulated in Table 2.

**Table 2:** Summary of the articles included for review CAP, community acquired pneumonia; SOFA, Sequential Organ Failure Assessment; ECMO, extracorporeal membrane oxygenation; NA, not announced.

Author (Year)	Year published	Study design	Sample size	Median age of patients in study population	Most common cause of ARDS in study population	MV duration median prior to ECMO (days)	SOFA score at ECMO starting	RRT % during ECMO	Duration of ECMO (Mean or Median)	Mortality rate	Key findings
Roch [13]	2014	Prospective observational study	85	47	CAP	2 (1–8)	9 (7–11)	48	9	56	The study suggests that factors like the patient's age, the SOFA score assessed within 3 hours prior to ECMO, and influenza pneumonia diagnosis are independently tied to the mortality rate in ARDS patients who are transferred to the ECMO center from distant hospitals.
Haneya [14]	2015	Retrospective observational study	262	49	Pneumonia	1 (1–3)	12 (8.8–15)	19.8	9	40	The study concludes that complications such as acute kidney injury (AKI) significantly contribute to mortality in severe ARDS patients. It was also found that the necessity for renal replacement therapy (RRT) before initiating ECMO, elevated lactate levels, and high blood transfusion requirements negatively affect the survival rate. However, the development of AKI during ECMO that requires RRT was not independently linked to the mortality rate.
Schmidt [15]	2015	Retrospective observational study	168	41	Bacterial pneumonia	2(1–6)	10 (8–13)	45	10	29	The study established that there is an association between reduced mortality and an early higher PEEP with MV when receiving ECMO support.
Burrell [16]	2017	Prospective observational study	262	49	NA	1(1–5)	12(8–15)	NA	8	22	The study observed a rapid decline in cytokine levels after the initiation of VV ECMO, suggesting this is related to the decrease in aggressive mechanical ventilation, which was the primary treatment change post-VV ECMO implementation. Elevated cytokine levels before and during VV ECMO were found to be linked to extra-pulmonary causes of ARDS, more invasive mechanical ventilation, and an increased risk of death.
Na [17]	2019	Retrospective observational study	487	58	Bacterial pneumonia	1(0–6)	11(8–14)	NA	8	62.2	The study suggested that patients with severe acute respiratory failure receiving ECMO support for an extended duration didn't exhibit worse short- and long-term survival rates compared to those on ECMO for 28 days or less. It emphasized that ECMO duration should not be a factor to determine treatment futility.
Lim [18]	2019	Retrospective observational study	335	55	Bacterial pneumonia	NA	8 (4–12)	NA	16	62.6	The study concluded that fem–jug and fem–fem configurations showed similar clinical outcomes in terms of short-term oxygenation in patients with ARDS on VV-ECMO. However, it was noted that the fem–jug configuration had a higher incidence of ECMO-related complications.
Galvagno [19]	2020	Retrospective observational study	194	42	Bacterial pneumonia	NA	11 (8–14)	51.3	19	24.7	The study did not find any significant correlation between obesity and increased mortality in patients requiring VV ECMO, hinting at the potential existence of the “obesity paradox” in this patient population.
Warren [20]	2020	Retrospective observational study	1205	44	Bacterial pneumonia	2 (1–5)	NA	NA	16	26	The study observed factors such as younger age, higher patient weight, asthma as presenting diagnosis, and less severe hypoxemia at the decision-to-cannulate point were associated with survival to ECMO ICU discharge in the cohort.
Seeliger [21]	2021	retrospective observational study	218	49	NA	NA	9(8–10)	NA	9	41.7	The study indicated that the institutional approach of high-dose heparinization during ECMO support was associated with lower rates of oxygenator changes and thromboembolic events, compared to low-dose heparinization.
Urner [22]	2022	Retrospective observational study	7345	59	COVID-19	NA	4(3–6)	10	13	50	The study found that ECMO was associated with a reduction in mortality by 7.1% compared with conventional mechanical ventilation without ECMO in patients with covid-19 associated acute respiratory failure. It also suggested that age, severity of hypoxaemia, intensity of mechanical ventilation, and duration of mechanical ventilation should be considered when deciding to initiate ECMO.
Warren [23]	2022	Retrospective observational study	847	43.6	Bacterial pneumonia	3(2–7)	NA	NA	16	24	The study suggested that patients with less severe hypoxemia who cannot be ventilated in a “lung-protective” fashion may derive a higher benefit from ECMO than those with refractory respiratory failure.
Guervilly [24]	2022	RCT	310	56	COVID-19	8(6–12)	NA	NA	NA	37	The study concluded that a multimodal ultra-lung-protective strategy including intermittent prone position was not associated with a decrease in the pulmonary and the systemic biotrauma compared with the lung-protective strategy of the EOLIA trial in severe

Discussion

The systematic review of 12 studies focused on the mortality rates and associated factors in patients with ARDS receiving ECMO therapy. The median age of study participants ranged from 41 to 59 years across the studies. The most common cause of ARDS reported in these studies was pneumonia, either community-acquired, bacterial, or due to COVID-19. The duration of mechanical ventilation (MV) prior to ECMO had a median ranging from 1 to 8 days. The SOFA score at the initiation of ECMO ranged from 8 to 12. Renal replacement therapy (RRT) percentages during ECMO, where reported, varied widely from 19.8% to 51.3%. The mean duration of ECMO, which was not consistently reported across all studies, ranges from 8 to 16 days.

Mortality Rates and Predictors

The mortality rates ranged between 22% and 62.6% in ARDS patients receiving ECMO therapy, with several factors emerging as potential predictors of mortality. The age of patients was identified as a significant factor, with older age being associated with increased mortality [13]. Similarly, Warren et al. found that a younger age at the time of ECMO ICU discharge was a positive survival indicator, emphasizing the critical role of patient age in determining ECMO therapy outcomes [20].

Impact of Comorbidities and Complications

Comorbidities and complications occurring during ECMO therapy were substantial factors influencing patient outcomes. Notably, acute kidney injury (AKI) was a significant complication associated with high mortality in patients with severe ARDS [14]. However, the development of AKI necessitating RRT during ECMO was not found to be an independent risk factor for mortality, indicating the need for further research to understand the complex interplay between AKI, RRT, and mortality in ARDS patients on ECMO. Interestingly, the study by Galvagno et al. did not find a significant correlation between obesity and increased mortality, suggesting the potential existence of the "obesity paradox" in ARDS patients on ECMO [19]. Other complications usually involved while on ECMO therapy include bleeding from the anticoagulation used for the ECMO circuit. A retrospective analysis of 132 patients showed a bleeding risk of 56.1% with a rate of 10 events per 100 days, which in turn showed a higher mortality at 90 days compared to those who did not bleed [25].

Treatment Considerations and Implications

Treatment-related factors also played a crucial role in patient outcomes. Higher PEEP with MV during ECMO support was associated with decreased mortality, underscoring the significance of appropriate ventilation management in these patients [15]. Burrell et al. reported a rapid decline in cytokine levels upon initiation of ECMO treatment, implying the potential anti-inflammatory benefits of ECMO, which may contribute to improved outcomes [16]. Interestingly, Na et al. found that a longer ECMO duration did not necessarily result in a poor prognosis, indicating that the duration of ECMO should not be used as a definitive marker for treatment futility [17]. Cannula configurations in patients with ARDS under VV-ECMO also played a role in determining clinical outcomes. Lim et al. found that fem-jug and fem-fem configurations showed similar clinical outcomes in terms of short-term oxygenation, but the fem-jug configuration had a higher incidence of ECMO-related complications [18]. Anticoagulation strategies during VV-ECMO support significantly influenced patient outcomes, with a high-dose heparinization approach linked to lower rates of oxygenator changes and thromboembolic events compared to low-dose heparinization [21].

Implications for COVID-19 ARDS Patients

For patients with COVID-19-associated ARDS, ECMO was associated with a reduction in mortality compared with conventional MV [22]. The study also emphasized the importance of factors like age, severity of hypoxemia, intensity of MV, and duration of MV when deciding to initiate ECMO. Furthermore, Warren et al. suggested that patients with less severe hypoxemia who cannot be ventilated in a “lung-protective” fashion may derive a higher benefit from ECMO than those with refractory respiratory failure [20,23]. Interestingly, Guervilly et al. concluded that a multimodal ultra-lung-protective ventilation strategy, including low tidal volume ventilation and prone positioning during VV-ECMO, may reduce biotrauma, potentially improving outcomes in severe ARDS patients [24].

Other Potential Factors Related to Mortality

Factors that are associated with complications while receiving ECMO can be potentially related to increased mortality. Complications such as bleeding, thrombocytopenia, neurological issues, and infections have been implicated in worse outcomes and long-term sequelae in survivors of ECMO therapy [25,26]. Patients can also experience refractory hypoxemia or concomitant cardiac failure despite receiving ECMO therapy which further lowers the survival rates. Prone positioning has been shown to improve oxygenation and also lower mortality in patients with ARDS on ECMO. A retrospective cohort study consulted by Giani et al. showed a significant reduction in mortality (30% vs 53%; p = 0.0241) with an odds ratio of 0.50; 95% confidence interval, but had longer ECMO duration (16 vs 10 days, p = 0.034) [27].

Prognostic Scores

Prognostic scores play a crucial role in predicting the mortality and outcomes in patients with ARDS who are on ECMO. One such commonly used score is the SOFA score, which helps predict ICU mortality based on the degree of dysfunction in six organ systems [28]. Studies have found an association between higher SOFA scores at the initiation of ECMO and increased mortality in patients with ARDS. Similarly, the ECMO Net Mortality Prediction (ENMP) model, a specific prognostic score developed for ECMO patients, has been shown to provide reliable predictions of hospital mortality and can be used for benchmarking performance across centers [29]. The RESP score, another predictive model, is useful for identifying patients with severe ARDS who would most likely benefit from ECMO therapy [30]. However, it's crucial to note that while these scores are helpful tools, they should not be used in isolation, and the decision to initiate ECMO should also consider various patient-specific factors and clinical judgments [9].

Limitations

The studies included in this systematic review are mainly observational studies, which create heterogeneity. The studies included in this systematic review varied in their design, study population, ECMO implementation and management protocols, and outcome definitions. This heterogeneity may have contributed to variations in reported mortality rates and might have affected the identification of factors associated with mortality. The review only included articles published within the last ten years and in the English language, which might have excluded relevant data from older studies or studies published in other languages. While this review aimed to identify factors associated with mortality in ARDS patients on ECMO, the secondary outcomes (like the cause of ARDS, SOFA scores at ECMO initiation, duration of ECMO, etc.) were inconsistently reported across studies. This inconsistency limited the ability to conclusively identify these factors.

## Conclusions

In conclusion, ARDS remains a critical health challenge with high mortality rates worldwide. ECMO has emerged as a potentially life-saving therapy for patients with severe ARDS who do not respond to conventional treatments. Despite recent advancements in technology and patient management, the optimal approach for ARDS patients on ECMO remains a matter of debate, with mortality rates exhibiting significant variability across different studies ranging from 22% and 62.6%. The variability of mortality can be explained by various factors, especially patient-related factors such as age, severity of hypoxemia, cause of ARDS, and development of organ damage like acute kidney injury. Also, ECMO-related factors like developing thrombocytopenia, bleeding from anticoagulation, and canula-related factors influence mortality rates. Ultimately, a comprehensive understanding of the factors affecting mortality rates in ARDS patients on ECMO can guide clinical practice, improve patient outcomes, and help reduce the global burden of this severe and life-threatening disease.
